# A single-cell and tissue-scale analysis suite resolves Mixl1’s role in heart development

**DOI:** 10.1016/j.isci.2025.112397

**Published:** 2025-04-10

**Authors:** Magdalena E. Strauss, Mai-Linh Nu Ton, Samantha Mason, Jaana Bagri, Luke T.G. Harland, Ivan Imaz-Rosshandler, Nicola K. Wilson, Jennifer Nichols, Richard C.V. Tyser, Berthold Göttgens, John C. Marioni, Carolina Guibentif

**Affiliations:** 1 European Molecular Biology Laboratory, European Bioinformatics Institute, Hinxton, Cambridge CB10 1SD, UK; 2 Department of Mathematics and Statistics, University of Exeter, Exeter EX4 4PY, UK; 3 Wellcome Sanger Institute, Wellcome Genome Campus, Hinxton, Cambridge CB10 1SA, UK; 4 Department of Haematology, University of Cambridge, Cambridge CB2 0AW, UK; 5 Cambridge Stem Cell Institute, University of Cambridge, Cambridge CB2 0AW, UK; 6 MRC Human Genetics Unit, Institute of Genetics and Cancer, University of Edinburgh, Edinburgh EH4 2XU, UK; 7 Institute Biomedicine, Department of Microbiology and Immunology, Sahlgrenska Center for Cancer Research, University of Gothenburg, 413 90 Gothenburg, Sweden

**Keywords:** Biocomputational method, Genomic analysis, Genomics

## Abstract

Perturbation studies using gene knockouts have become a key tool for understanding the roles of regulatory genes in development. However, large-scale studies dissecting the molecular role of development master regulators in every cell type throughout the embryo are technically challenging and scarce. Here, we systematically characterize the knockout effects of the key developmental regulators *T/B*rachyury and *Mixl1* in gastrulation and early organogenesis using single-cell profiling of chimeric mouse embryos. For the analysis of these experimental data, we present COSICC, an effective suite of statistical tools to characterize perturbation effects in complex developing cell populations. We gain insights into *T*’s role in lateral plate mesoderm, limb development, and posterior intermediate mesoderm specification. Furthermore, we generate *Mixl1*^−/−^ embryonic chimeras and reveal the role of this key transcription factor in discrete mesoderm lineages, in particular concerning developmental dysregulation of the recently identified juxta-cardiac field.

## Highlights

• COSICC enables multi-layered statistical analysis of gene perturbation effects
• COSICC addresses compositional bias in single-cell profiling of development defects
• *T* plays a key cell-autonomous role in intermediate mesoderm and limb bud development
• *Mixl1* depletion impairs juxta-cardiac field, epicardium, and cardiac development

## Graphical abstract

**Figure:**
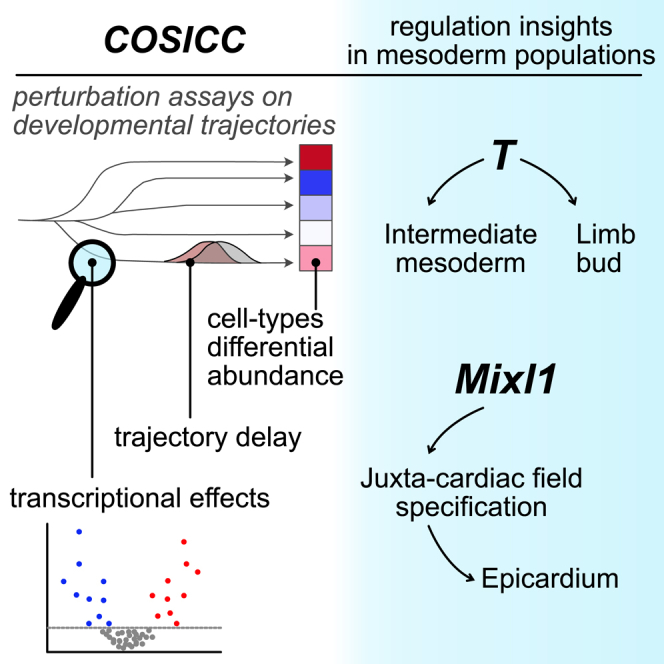

## Introduction

Using CRISPR knockouts in conjunction with a single-cell transcriptomic readout has become an important tool for understanding gene function. Although a number of studies have focused on the development of experimental and analysis techniques for large-scale screens, where up to thousands of genes are knocked down in cell lines,^1^ others have focused on smaller scale knockout analysis in more complex settings, such as mouse models^2^ or organoids.^3^

**Figure 1:**
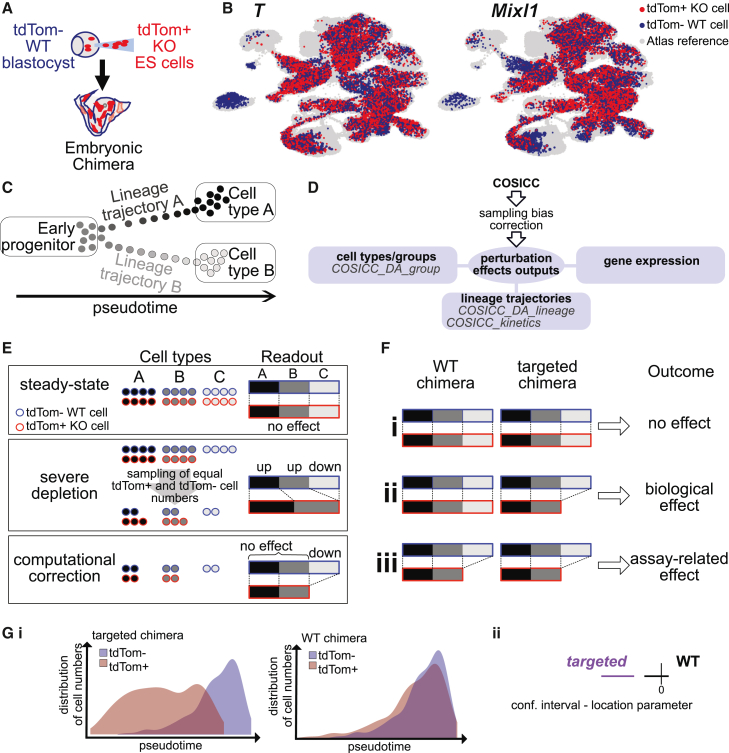
Overview of chimeric mouse embryos and COSICC framework (A) Chimeric embryos were created by injecting mutant tdTom^+^ cells into wild-type tdTom^−^ (WT) embryos at the blastocyst stage. (B) Chimeric mouse embryos mapped to the extended mouse gastrulation atlas (reference atlas cells in gray, tdTom^+^ cells in red, tdTom^−^ cells in blue). (C) Illustration of lineages. (D) Overview of COSICC. (E) Correction of sampling bias. Top: cell counts from an embryo without perturbation effect, with tdTom^+^ cells in red and tdTom^−^ cells in blue. Middle: depletion of one cell type only; this depletion is unknown before data analysis; therefore, a fixed proportion of cells with fluorescent markers is sampled, leading to cell types seemingly enriched for knockout cells. Bottom: computational correction. (F) Top: no change in cell-type abundance for either WT or targeted chimeras. Middle: reduction in abundance of tdTom^+^ cells, but no (or less) reduction for WT, for targeted chimeras. Bottom: similar levels of reduction in cell-type abundance in tdTom^+^ cells for WT and targeted chimeras. (G) Illustration of COSICC_kinetics output (i) and statistical assessment of perturbation induced delay (Wilcoxon rank-sum test, Methods, (ii)). See also Figures S1 and S7.

Within the context of such highly informative lower throughput model systems, the generation of embryonic chimeras, where mutant cells are injected into wild-type (WT) embryos at the blastocyst stage, is a powerful tool to study the function of essential developmental transcription factors^4^^,^^5^^,^^6^ (Figure 1A). In the resulting chimeras, WT cells develop normally, establishing signaling gradients necessary for the embryo to develop, whereas the effects of the knockout can be observed by studying the descendants of the injected mutant cells. Chimeras can therefore reveal the cell-autonomous role of essential genes, where a full knockout would lead to embryonic lethality and gross developmental malformations. Furthermore, single-cell profiling for chimeras enables the comprehensive study of knockout effects well beyond differences in organ contribution (Figures 1B, S1A, and S1B).

Although powerful, single-cell chimera studies are challenging to interpret, with sampling biases and other technical factors potentially confounding results. In particular, the systematic assessment of the impact of knockouts on lineage progression, gene expression, and the interaction of these effects remains challenging. To address these problems, we herein present COSICC (comparative analysis of single-cell RNA-sequencing data for complex cell populations), a set of computational tools specifically designed for the analysis of perturbations in complex developing cell populations compared to control experiments and therefore ideally suited to chimera studies. We applied this approach to dissect the role of two key gastrulation regulators, where conventional knockout embryos are embryonic lethal during organogenesis. We first validated COSICC using previously published data from chimeric *T/Brachyury*^−/−^ embryos, where *T* had been shown to regulate the egress of posterior mesoderm in the late stages of gastrulation, with *T*^−/−^ primitive streak cells contributing to an accumulated cell population with neuromesodermal progenitor transcriptional signature.^4^ We then generated *Mixl1*^−/−^ chimeric mouse embryos, which we analyzed using single-cell RNA sequencing (scRNA-seq) and revealed, through application of COSICC, the molecular and tissue-scale consequences of loss of function for this key developmental regulator at whole embryo scale. *Mixl1* is the only characterized mammalian member of the *Mix/Bix* transcription factor family, first identified in Xenopus as regulating mesendoderm development.^7^ Although *Mixl1*^−/−^ mouse embryos show severe developmental defects and early embryonic lethality, in a previous chimera assay *Mixl1*^−/−^ cells only showed contribution impairment to posterior gut tissues,^8^ whereas in the present study we characterize the molecular effects of *Mixl1* knockout throughout all embryonic lineages present at early organogenesis.

## Results

### COSICC leverages single-cell profiling to analyze developmental perturbations

We considered two independent chimera datasets, both generated at embryonic day (E) 8.5 of mouse development: (1) a previously published dataset that studied the cell autonomous function of *T* for validation of our analysis suite and (2) a newly generated dataset created to study the role of *Mixl1* in early organogenesis. Additionally, we used data from a control experiment, where WT cells were injected at the blastocyst stage to create control chimeras; this was also profiled at E8.5. In all chimeras, we define the progeny of injected cells as tdTomato-positive cells (tdTom^+^; Figures 1A and 1B), since the injected cells have constitutive expression of the fluorescent marker tdTomato. The WT (host) cells lack tdTomato expression and are referred to as tdTomato negative (tdTom^−^).

Profiling the chimeric embryos using scRNA-seq, in conjunction with reference single-cell atlases of early development, opens exciting possibilities for studying the effects of perturbations during cellular diversification (Figures 1B and S1A–S1C). Importantly, in the mouse embryo dataset used as a reference framework for our analysis of embryo chimeras, developmental trajectories were inferred by combining the Waddington optimal transport (Waddington-OT) matrices^9^ (STAR Methods) with probabilistic mixture modeling. We can thus explore whether the perturbed cells are depleted or enriched in certain cell types or in lineage trajectories leading to each cell type and assess the impact of the perturbation on developmental progression along specific lineage trajectories (Figure 1C).

Our COSICC analysis framework (Figure 1D) evaluates knockout effects on cell type or cell group abundance (COSICC_DA_group), lineage trajectory abundance (COSICC_DA_lineage), and developmental delay along lineage trajectories (COSICC_kinetics). COSICC addresses two specific challenges arising in the study of perturbations in complex systems. First, in an experiment where a fixed total number of cells are collected, if the perturbation leads to a strong reduction of some cell types or groups of cells, then the unaffected cell types/groups will appear as if they were enriched in the perturbed cell population (Figure 1E). COSICC_DA_group and COSICC_DA_lineage include a sampling bias correction approach to address this problem (Figure 1E; STAR Methods), based on sub-sampling with the assumption that most groups are not affected as a result of the knockout. It should be noted that the same type of sampling bias also occurs in any perturbation experiment with single-cell analysis readouts, where the number of sampled cells is pre-determined based on technical constraints. Second, there are experimental effects inherent to a chimera-based assay that may confound biological insights. All analyses were thus framed in comparison with an external control dataset, the WT chimeras introduced earlier (Figures S1C and S1D). Chimera generation may lead to transcriptional differences between tdTom^+^ and tdTom^−^ cells, due to, for instance, *ex vivo* culture applied to tdTom^+^ cells prior to injection, which can affect their transcriptome even in the absence of genetic editing. COSICC therefore performs DA testing with reference to the external control, thus accounting for experimental effects (Figure 1F).

A framework to consider the external control is also included in COSICC_kinetics (Figure 1G), which identifies dynamic shifts in lineage development by testing whether developmental progression along a lineage trajectory has been delayed or accelerated for the knockout cells (STAR Methods). Finally, we applied to each cell type a mixed effects model approach^10^ to test for differential gene expression (DE), again contrasting to external controls, and also accounting for batch effects across different pools of chimeric mouse embryos (Figure S2, STAR Methods).

In addition to the development of COSICC, our analysis framework uses a substantially extended reference atlas of mouse gastrulation^11^ compared to the reference atlas used in previous work.^6^ This extended mouse gastrulation atlas contains four additional time points between E8.5 and E9.5, four times as many cells as the previous reference, as well as updated cell type annotations, thereby more completely representing early mouse organogenesis (Figures 1B and S1A–S1C). Using this extended reference dataset allowed us to identify cell type, stage, and pseudotime of chimeric cells that, in spite of being collected at E8.5, were more similar to cells present in later stage embryos due to asynchrony in developmental progression within embryo litters^12^ or because of faster progression along a developmental lineage trajectory (E8.75 and later; Figure S1D).

### Characterization of the impact of *T* knockout on embryonic development reveals its cell-autonomous role for intermediate mesoderm as well as limb development

**Figure 2:**
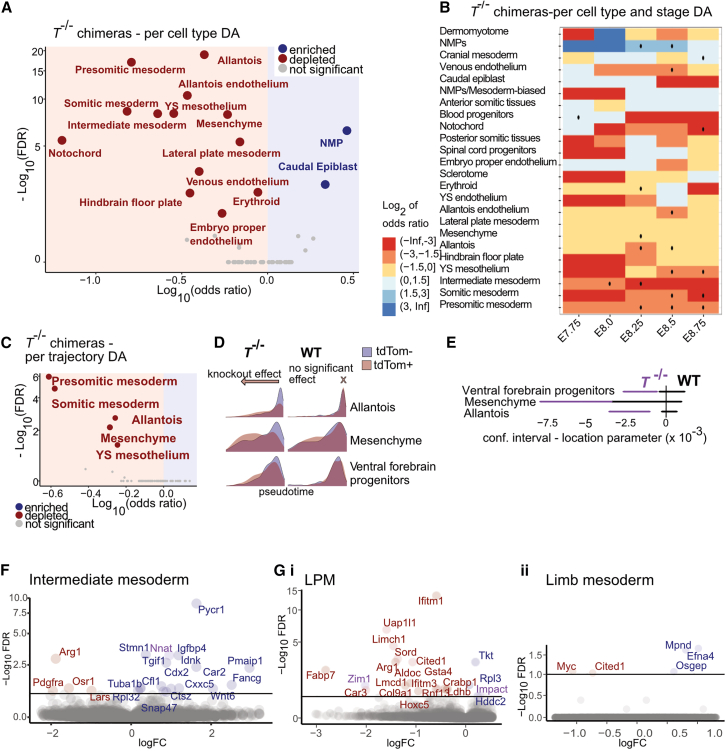
COSICC confirms existing results and reveals mechanistic insights into the role of *T* in the development of discrete mesodermal populations (A) DA at the cell-type level for *T*^−/−^ chimera knockout cells (COSICC_DA_group). (B) Per-stage DA at cell-type level. Significance at FDR<0.1 is indicated by a dot. Cell types found to be significantly enriched or depleted (A) and related cell types are shown. (C) DA of Waddington-OT trajectories leading to the listed cell types at E9.25 (COSICC_DA_lineage). (D) Pseudotime distributions of tdTom^+^ (red) and tdTom^−^ (blue) cells within *T*^−/−^ and WT chimeras. (E) Confidence interval for location parameter of Wilcoxon rank-sum test (COSICC_kinetics). Confidence intervals for *T*^−/−^ chimeras (purple bars) overlapping neither 0 nor the confidence intervals for WT chimeras (black bars) imply significant developmental delay (Figure 1G, ii). (F) Volcano plot illustrating statically differentially expressed genes for intermediate mesoderm. (G) Volcano plot illustrating statically differentially expressed genes for LPM (i) and limb mesoderm (ii). (F and G) Imprinted genes, taken from Supplementary Data 1 of Santini et al.^57^ are in purple. The x axis represents the log2-fold change for differential expression contrasted with the WT chimeras. YS, yolk sac. (A–C) Odds ratio and FDR values calculated using Fisher’s exact test (see STAR Methods). See Table S2 for cell numbers for each stage (mapped from the extended mouse gastrulation atlas), cell type, and sample. See also Figures S3, S4, and S6.

We used all elements of COSICC (Figure 1D) to obtain a comprehensive view of the effects of *T* knockout. First, the cell-type level analysis of *T*^−/−^ chimeras with COSICC_DA_group confirmed previous results,^4^ with depletion of somitic mesoderm, presomitic mesoderm, intermediate mesoderm, and notochord in the mutant cells and enrichment for neuromesodermal progenitors (NMPs; Figure 2A). In addition, we observed a broad depletion of other mesodermally derived cell types including endothelial cell types, namely embryo proper endothelium, venous endothelium, and allantois endothelium, as well as mesenchyme, yolk sac mesothelium, and allantois. We also observed enrichment of caudal epiblast cells, which was only present at earlier time points in WT embryos, suggesting a developmental delay of mutant cells and impaired gastrulation as observed previously.^4^^,^^13^ Additionally, our pipeline facilitated assessment of time-dependent cellular abundance changes, as seen, for example, for blood progenitors and cranial mesoderm, which showed significant changes in relative abundance across different time points (Figure 2B). We also noted that accounting for the bias illustrated in Figure 1E avoids incorrect conclusions about cell types being enriched for knockout cells (Figure S3A).

Second, at the level of lineage trajectories, results from COSICC_DA_lineage were broadly concordant with the cell-type level results, with *T*^−/−^ cells significantly depleted in trajectories toward somitic mesoderm, presomitic mesoderm, allantois, mesenchyme, and yolk sac mesothelium (Figures 2C and S3B).

Third, among the trajectories containing sufficient cells to assess lineage progression in both mutant and WT fraction (Figures S3C and S4), COSICC_kinetics revealed a delay for both mesenchyme and allantois lineage development as a result of *T* knockout (Figures 2D and 2E). We also observed a delay in the trajectory leading to an ectodermal cell type, the ventral forebrain progenitors. Interestingly, our analysis shows a depletion of the mesenchyme cell type as well as a delay in its lineage development (Figures 2D and 2E), which links to an association between overexpression of *T* and changes in epithelial-to-mesenchymal transition reported in cancer.^14^^,^^15^^,^^16^ Further, the depletion of the allantois cell type and lineage trajectory, and the developmental delay along that lineage trajectory observed in tdTom^+^ *T*^−/−^ cells (Figures 2A–2D), are in line with well-documented allantois defects for *T* knockout mice and chimeric embryos,^17^ giving confidence in the performance of COSICC.

We noted a striking effect of *T* knockout on the intermediate mesoderm cell type, significant at the cell-type level as a whole (Figure 2A) and in particular for cells mapping to E8.0 and E8.25 time points (Figure 2B). We further explored gene deregulation in this cell type by performing DE analysis (Figure 2F; Table S1) and showed significant downregulation of the intermediate mesoderm master regulator *Osr1* as well as mesenchymal marker *Pdgfra*. Conversely, we see upregulation of epiblast genes *Pmaip1*^18^ and *Tgif1*^19^ and caudal mesoderm marker *Cdx2*, along with deregulation of the Wnt pathway (with upregulation of *Wnt6* ligand as well as Wnt signaling inhibitor *Igfbp4*^20^). Metanephric mesenchyme was shown to derive from caudal mesoderm precursors expressing high levels of *T*, through an Osr1-expressing posterior intermediate mesoderm state.^21^ With a drastic cell-autonomous effect of *T* knockout on intermediate mesoderm cells, our data thus suggest that *T* plays a pivotal role in the regulation of posterior derivatives of intermediate mesoderm relevant for kidney development, likely through direct modulation of *Osr1* expression.^22^

We also observed significant depletion of *T*^−/−^ cells in lateral plate mesoderm (LPM). Since *T* is expressed in the LPM at early stages of the limb mesoderm trajectory (Figure S3D), we decided to investigate the impact of knocking out *T* on limb mesoderm development from LPM, given the impaired forelimb bud formation in *T* mutant embryos,^23^ where the underlying (*T*-dependent) mechanisms regulating forelimb formation has remained unclear.^24^ We explored this further by performing DE analyses for both LPM and limb mesoderm (Figure 2G; Table S1). Interestingly we observed downregulation of *Cited1* in both LPM and limb mesoderm *T*^−/−^ cells. Although *Cited1* expression has been reported in defined limb mesoderm subsets,^25^^,^^26^ its role in limb development remains unclear—our results suggest it may act downstream of *T* already within the LPM limb precursor cells. In LPM *T*^−/−^ cells, we also observed downregulation of *Hoxc5*, member of the Hox5 family, directly implicated in forelimb development.^27^ Finally, limb mesoderm *T*^−/−^ cells also displayed downregulation of *Myc*, a key gene in limb development.^28^

Taken together, our results indicate that beyond its previously documented role in orchestrating embryo axial elongation and somitogenesis, *T* also has important roles in cell-type specification within other mesodermal populations, specifically intermediate mesoderm and limb bud development.

### *Mixl1* knockout depletes epicardium and specific cardiac progenitor populations

Having validated COSICC on a previously published *T*^−/−^ chimera dataset, where we identified a cell-autonomous role of T for intermediate mesoderm specification and forelimb development, we proceeded to apply the same framework to a newly generated chimera dataset. We decided to investigate the role of another gastrulation master regulator, the transcription factor *Mixl1*. Similarly to *T* knockout embryos, *Mixl1* mutants also show shortening of the anterior-posterior axis; however, in addition they display disorganization of anterior structures (absence of a heart tube and abnormal headfolds) and severe gut developmental defects.^8^ However, the molecular role of *Mixl1* within these cell populations has not been investigated. To systematically assess the embryo-wide effects of *Mixl1* knockout, we generated *Mixl1*^−/−^ chimeras and processed the resulting scRNA-seq data using COSICC. *Mixl1*^−/−^ tdTom^+^ mouse embryonic stem cells were generated using CRISPR/Cas9 to induce frameshift mutations in exon 2 of the *Mixl1* locus, thereby causing an early stop codon and functional inactivation of the gene and its homeobox domain (Figure S5A). Two independent *Mixl1*^−/−^ tdTom^+^ clones were injected into WT blastocysts to generate embryonic chimeras, and three independent pools of E8.5 *Mixl1*^−/−^ chimeras were collected, containing three and six embryo chimeras from clone 1, respectively, and four embryo chimeras from clone 2. Single-cell suspensions were generated from each pool of embryos independently, and cells were sorted by flow cytometry based on the tdTom reporter and analyzed by scRNA-seq (see STAR Methods).

**Figure 3:**
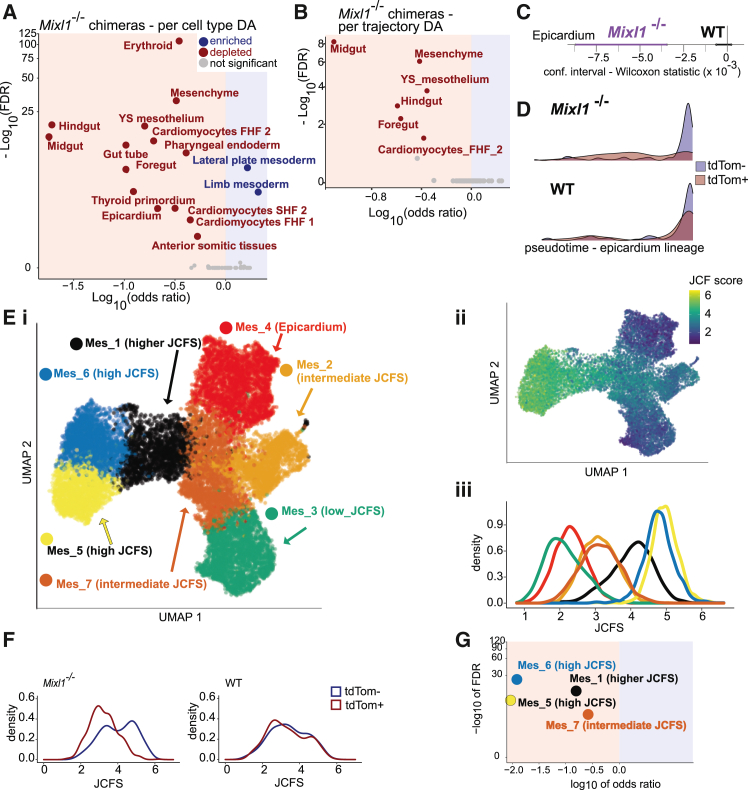
COSICC reveals mechanistic insights into epicardium development (A) DA at the cell-type level for *Mixl1*^−/−^ chimera knockout cells (COSICC_DA_group). (B) COSICC_DA_lineage. (C) Confidence interval for location parameter of Wilcoxon rank-sum test (COSICC_kinetics). (D) Pseudotime distributions tdTom+ (red) and tdTom− cells (blue) within *Mixl1*^−/−^ and WT chimeras for the epicardium lineage. (E) Sub-clustering of mesenchyme. (i) Uniform manifold approximation and projection (UMAP) coordinates recomputed for the cells annotated as epicardium or mesenchyme in the reference dataset. (ii) UMAP colored by JCF score (JCFS). (iii) Density plot of JCFS split by cluster. Clusters in (i) were labeled according to their levels of JCFS. Cluster Mes_4 mostly comprised the cells labeled as epicardium in the reference dataset. (F) Distribution of JCFS for *Mixl1* and WT chimeras across all cells from the mesenchyme cell type shows a depletion of JCF signature for the *Mixl1*^−/−^ cells. (G) COSICC_DA_group reveals strong depletion for *Mixl1*^−/−^ cells for the clusters with high JCFS. To perform the normalizing step (Figure 1E), we ran COSICC_DA_group across all cell types, with mesenchyme replaced by the new subclusters, and plotted results for the mesenchyme subclusters. YS: Yolk Sac. See Table S2 with *n* values corresponding to the number of cells for each cell type and sample. See also Figures S3–S6.

Using COSICC_DA_group and COSICC_DA_lineage in *Mixl1*^−/−^ chimeras, we observed a broad effect on LPM and its derivatives: a depletion of cell types and lineages for cardiomyocytes of both first and second heart fields as well as epicardium and erythroid cells and an increased representation of LPM and limb mesoderm (Figures 3A, S5B, and S5C). Collectively therefore, these observations amount to a depletion of splanchnic mesoderm with simultaneous enrichment of somatic mesoderm. Although *Mixl1*^−/−^ mutants have been previously shown to have defective cardiac development,^8^^,^^29^ the molecular role of *Mixl1* for cardiac cell-type induction has remained elusive.

In addition to the impact on heart development, *Mixl1* knockout mice are known not to form a hindgut.^8^ Consistently, in the *Mixl1*^−/−^chimeras, hindgut and midgut were fully depleted at cell type level, whereas foregut, gut tube, pharyngeal endoderm, and thyroid primordium were all partially, but substantially, depleted (Figures 3A and S5B).

Proceeding to investigate lineage trajectory development, note that COSICC_DA_lineage was not applicable to gut tube and pharyngeal endoderm, as there were too few cells at the final, E9.25, time point of the reference dataset (STAR Methods, Figures S3C and S4). Consistent with the *T*^−/−^ chimera analysis, all cell types in the *Mixl1*^−/−^ chimera that were depleted at the lineage trajectory level were also depleted at the cell-type level (Figures 3B and S5C), in line with a general concordance between cell-type differential abundance and changes in contribution to corresponding lineage trajectories. Finally, COSICC_kinetics revealed developmental delay for the epicardium lineage (Figures 3C and 3D). In addition to the depletion of cardiac cell types (Figures 3A and 3B) and substantial delay along the epicardium lineage trajectory (Figures 3C and 3D), we observed a depletion of cells assigned to the mesenchyme cell type and lineage (Figures 3A and 3B), a cell type that we found to be part of the epicardium trajectory (Figure S5D). Furthermore, we found a substantial number of differentially expressed genes for mesenchyme (Figure S5E; Table S1).

Prompted by the developmental delay in epicardium development, the impact on cardiac cell types, and the deregulation of the mesenchyme cell type on the epicardium trajectory together with recent reports suggesting previously unrecognized cardiac progenitor populations,^30^ we explored further the effects on cardiac development of *Mixl1* knockout. In particular, the gene deregulation of mesenchyme led us to investigate whether we could identify precursors of the epicardium within the population labeled as mesenchyme in the extended mouse gastrulation atlas. Indeed, sub-clustering the mesenchyme cell type (Figure 3E) revealed transcriptionally defined subsets, segregated by the expression of markers for a recently identified epicardial and cardiomyocyte progenitor population termed the juxta-cardiac field (JCF).^30^ This observation is consistent with the notion that cells annotated as mesenchyme may comprise JCF cells that would constitute putative progenitors for the cardiac cell types affected in *Mixl1*^−/−^chimeras. Indeed, the average expression of JCF markers (JCF score, JCFS; see Methods) was downregulated in *Mixl1*^−/−^ cells mapping to the mesenchyme cell type (Figure 3F; *p* value< 2.2e^−16^ for Wilcoxon rank-sum test), and clusters with high JCFS were strongly depleted in *Mixl1*^−/−^chimeras (Figure 3G). Altogether, this suggests a previously unknown role for *Mixl1* in the onset of a JCF program, impacting the downstream development of cardiac cell types, including the epicardium.

Using COSICC, we can easily compare effects of different perturbations if they can be mapped to the same reference framework, in this case the extended mouse gastrulation atlas. As mentioned before, both *T* and *Mixl1* have been reported as major mesoderm regulators.^4^^,^^8^^,^^31^^,^^32^ Comparing embryo-wide effects of both knockouts, we observed that the two sets of chimeras generally had distinct phenotypes (Figure S6). Interestingly, LPM was depleted in *T*^−/−^ chimeras and enriched in *Mixl1*^−/−^ chimeras (Figure S6). Moreover, both chimeras displayed distinct defects in LPM derivatives, with a depletion of endothelial cells in *T*^−/−^ chimeras compared to defective development of cardiac tissues in *Mixl1*^−/−^ chimeras. This suggests independent roles of the two transcription factors in the development of populations derived from LPM. Alternatively, the population annotated as LPM in the atlas may also comprise heterogeneous progenitors with restricted fates, where *Mixl1* and *T* regulate distinct gene networks.

## Discussion

Recent years have seen an increasing number of large-scale perturbation experiments^33^ in complex systems *in vivo*^2^^,^^34^ and *in vitro*,^3^ as well as a growing wealth of reference atlases being generated,^35^^,^^36^ including in the context of international consortia,^37^^,^^38^ along with the emergence of single-cell analysis of patient material.^39^^,^^40^ Principled analysis of large-scale complex perturbation data will be instrumental to harness such datasets in the context of increasingly granular single-cell reference atlases, leading to biological and clinical insights.

Single-cell readouts for perturbation experiments in complex systems allow insights into molecular, cellular, and tissue-scale processes across a wide range of cell types and lineages. Here, we used scRNA-seq data generated from chimeras to study the role of the transcription factors *Mixl1* and *T* across all lineages in the developing mouse embryo. For this purpose, we developed a set of computational tools, COSICC, which enabled us to tackle the particular challenges of complex perturbation data. Our experimental and computational approach allowed the identification of affected lineage trajectories, such as limb mesoderm and intermediate mesoderm for *T* and epicardium for *Mixl1*.

The COSICC framework is also relevant to applications outside of the specific use case of chimeric embryos in three ways. First, chimeric organoid systems, where mutant cells are cultured in the context of WT aggregates, are emerging as promising alternatives to study genetic perturbations, including malignancies, *in vitro.*^41^^,^^42^^,^^43^^,^^44^ The entire suite of COSICC will be a highly effective way of analyzing single-cell studies in these types of systems. Second, the sampling bias correction step introduced for unbiased assessment of differential abundance at the level of cell type, cell group, or lineage trajectory is widely applicable to the detection of the effect of CRISPR or drug perturbation on cell-type composition for any experiment with a single-cell readout, e.g., in organoids, mouse models, or non-model organisms,^45^ and has the potential to prevent future false-positive results of overrepresentation of cell types in any such system. Third, while COSICC was designed for chimeric systems and development, where we see the strongest potential of the method and where there has been exciting experimental developments with chimeric organoids, we demonstrated the wider relevance of the proposed computational analysis methods to general perturbation and disease scRNA-seq data by applying it to a study of AML patient data^46^ (Figure S7).

COSICC is a highly flexible framework. We used mutual nearest neighbors^47^ to map chimera data to the reference dataset, for consistency with the batch correction method previously applied to our reference dataset.^11^ However, COSICC may be used with any mapping,^48^^,^^49^^,^^50^ cell lineage estimation,^51^ and pseudotime/cell ordering method. Instead of pseudotime, COSICC could alternatively be combined with the transcriptional rank of the individual embryo in the reference dataset to which the chimera cell is most similar.^5^ COSICC is a cell-specific coordinate-based approach, locating each individual cell within a framework of precomputed scores on a reference dataset (e.g., the extended mouse gastrulation dataset). This includes lineage scores (e.g., Waddington-OT) and lower-dimensional representations (e.g., diffusion maps). This coordinate-based approach, where each cell is associated with its own coordinates, differs from neighbourhood-based approaches that look for small sets of similar cells between a query and a reference dataset.^36^^,^^52^ This cell-specific coordinate-based strategy maintains the high granularity of the single-cell approach and provides a location of the cell in terms of the normal development of a reference atlas, along a specific direction (e.g., lineage trajectory).

The application of COSICC to the *T*^−/−^ chimera dataset validated our method by reproducing results from previous analyses.^4^ Due to better cell-type identification thanks to the extended reference atlas, we detected additional significant effects of *T* knockout on notochord (Figure 2A), strongly supported by the literature.^17^^,^^31^^,^^32^ Finally, our chimera-based experimental approach followed by single-cell transcriptional readout and systematic analysis using the COSICC workflow enabled the dissection of the cell-autonomous role of the *T* transcription factor within discrete mesodermal populations, shedding light on previously unknown molecular roles of *T* in intermediate mesoderm and limb development.

We then generated an additional chimera dataset targeting another key gastrulation regulator, *Mixl1*. In addition to the expected strong depletion of definitive endoderm tissues,^8^ we noted a marked impact on cardiac lineages, in particular a depletion of specific groups of precursor cells to epicardium. Indeed, *Mixl1*-KO mice show severe cardiac malformations,^29^ and epicardium plays an important role in coordinating the development of the other heart tissues, namely through modulation of the *Wnt* pathway.^53^ Furthermore, *Mixl1* has recently been implicated in an LPM inducing regulatory transcription factor network conserved in chordates,^54^ but based on the studied enhancer region, this previous study did not extend to mammals. Our observations of depleted splanchnic mesoderm (cardiac tissues) and enrichment of somatic mesoderm (limb) suggests that *Mixl1* may also have a major role in balancing lineage propensity within LPM precursors in mammalian embryos. Finally, our systematic analysis also allowed us to refine cell typing in the reference dataset. We found that the general impact of *Mixl1* knockout on cardiac development is reflected by the failure of *Mixl1* knockout cells to develop a JCF signature. This observation has led to identification of cell subsets with a high JCF signature within the mesenchyme cell type in the extended mouse gastrulation atlas.

### Limitations of the study

All our computational analysis steps, and in particular finer qualitative analyses, require sufficient cell numbers of perturbed and WT cells mapping to the specific cell types/lineage trajectories (see Figures S3C and S4). It should be noted that this is not a problem of the COSICC method per se, as any method based on the concepts of rigorous statistical inference will detect significant differential abundance or expression only in the presence of sufficient evidence, i.e., a large enough sample size. In fact, this general problem can be limiting for the analysis of rare populations or in settings where there is limited access to sufficient cell numbers. *Mixl1* and *T* knockout lead to strong depletion of several cell types, which means that for the respective lineage trajectories we were not able to use COSICC_kinetics to identify potential lags in development or test for differential gene expression, as this would have required the presence of both mutant and WT cells mapping to those cell types. In that sense, the present study informs future work aiming at dissecting the molecular role of these master regulators in the development of cell types found here to be completely depleted at E8.5, for instance to design experiments where embryos are collected at earlier time points in order to capture the affected developmental precursor of interest.

## Resource availability

### Lead contact

Further information and requests for resources and reagents should be directed to and will be fulfilled by the lead contact, Carolina Guibentif (carolina.guibentif@gu.se).

### Materials availability

Wildtype tdTomato and mutant *Mixl1*^−/−^ mESC lines are available upon request.

### Data and code availability

• Single-cell RNA-seq data for the *Mixl1* chimeras have been deposited at Arrayexpress and are publicly available. Accession numbers are listed in the key resources table.
• The COSICC R package (https://github.com/MarioniLab/COSICC) and all original analysis code in R (https://github.com/MarioniLab/analysis_chimera_data) have been deposited on Github and are publicly available (https://doi.org/10.5281/zenodo.15008255^55^). Count data for *T*^−/−^ and WT chimeras was obtained from,^4^ via the *MouseGastrulationData* Bioconductor package.^56^ Reference embryo data, including dimensionality reduction and Waddington-OT matrices, were obtained from a previous study.^11^
• Any additional information required to reanalyze the data reported in this paper is available from the lead contact upon request.

## Acknowledgments

We thank William Mansfield at the Wellcome-MRC Cambridge Stem Cell Institute animal facility for blastocyst injections, the Flow Cytometry Core Facility at the Cambridge Institute for Medical Research for cell sorting, Katarzyna Kania and the CRUK-CI genomics core for preparing the 10X libraries and for sequencing, and Jonathan Griffiths for comments on the manuscript. M.E.S. is supported by the 10.13039/100010269Wellcome Trust (220442/Z/20/Z). C.G. was funded by the 10.13039/501100004359Swedish Research Council (2017-06278) and by a Swedish Childhood Cancer Fund position grant (TJ2021-0009). M.-L.N.T. was funded by a Herchel Smith PhD Fellowship in Science. S.M. was funded by a 10.13039/501100000274British Heart Foundation PhD Studentship. J.B. was funded by a 10.13039/100010269Wellcome Trust PhD studentship. L.T.G.H. was funded by a 10.13039/100004440Wellcome Early-Career Award (226309/Z/22/Z). R.T. was funded by the British Heart Foundation Cambridge Centre of Research Excellence (RE/18/1/34212) and core support from 10.13039/100004440Wellcome to the Wellcome-MRC Cambridge Stem Cell Institute. Work in the Göttgens group is supported by 10.13039/100004440Wellcome, 10.13039/501100015570Blood Cancer UK, 10.13039/501100000265MRC, and 10.13039/501100000289CRUK and by core support grants from Wellcome to the Wellcome-MRC Cambridge Stem Cell Institute. This work was funded as part of a 10.13039/100004440Wellcome grant (220379/B/20/Z) awarded to B.G. and J.C.M. For the purpose of open access, the authors have applied a Creative Commons Attribution (CC BY) licence to any Author Accepted Manuscript version arising from this submission.

## Author contributions

M.E.S. analyzed the *Mixl1* embryonic chimera dataset, wrote all code, implemented COSICC, and applied it to all datasets presented; M.-L.N.T. performed *Mixl1* chimeric embryo dissections and generated the scRNA-seq dataset; C.G. generated the *Mixl1* knockout ESC lines; S.M. genotyped *Mixl1* ESC knockout clones; J.B. assisted with embryo dissections; N.W. assisted in scRNA-seq data generation; M.E.S, C.G., and J.C.M. interpreted the results with input from L.T.G.H., I.I.-R., M.-L.N.T., R.T., and B.G.; I.I.-R. provided processed data and Waddington-OT analysis for the extended mouse gastrulation atlas; M.E.S. wrote the first draft of the manuscript; M.E.S., C.G., L.T.G.H., M.-L.N.T., R.T., B.G., and J.C.M edited the manuscript; J.N., B.G., J.C.M., and C.G. supervised the study.

## Declaration of interests

J.C.M. has been an employee of Genentech since September 2022, and I.I.-R. is an employee of Altos labs. The remaining authors declare no competing interests.

## STAR★Methods

### Key resources table

**Table:** 

REAGENT or RESOURCE	SOURCE	IDENTIFIER
**Chemicals, peptides, and recombinant proteins**		

Proteinase K	Sigma	Sigma
Phusion High-Fidelity DNA Polymerase	NEB	M0530S
Lipofectamine 3000 Transfection Reagent	ThermoFisher Scientific	L3000008
TrypLE Express dissociation reagent	Thermo Fisher Scientific	12604013

**Critical commercial assays**		

Nextera XT Kit	Illumina	15052163
Chromium Single Cell 3ʹ GEM, Library & Gel Bead Kit v3	10X Genomics	PN-1000075
Chromium Chip B Single Cell Kit	10X Genomics	PN-1000073

**Deposited data**		

E8.5 *T*^-/-^ chimeras scRNAseq data	Guibentif et al.^4^	ArrayExpress: E-MTAB-8811
E8.5 WT chimeras scRNAseq data	Guibentif et al.^4^	ArrayExpress: E-MTAB-8812
extended mouse gastrulation scRNAseq raw data	Imaz-Rosshandler et al.^11^	ArrayExpress: E-MTAB-11763
extended mouse gastrulation scRNAseq processed data, including metadata and WOT trajectory inference	Imaz-Rosshandler et al.^11^	https://marionilab.github.io/ExtendedMouseAtlas/
E8.5 *Mixl1*^-/-^ chimeras scRNAseq data	This paper	ArrayExpress: E-MTAB-13409
GRCm38 Mouse reference genome Build 38	Genome Reference Consortium	https://www.ncbi.nlm.nih.gov/datasets/genome/GCF_000001635.20/
Annotated AML data	Van Galen et al.^46^	GEO: GSE116256

**Experimental models: Cell lines**		

TdTomato-expressing mouse embryonic stem cells	Pijuan-Sala et al.^6^	N/A

**Experimental models: Organisms/strains**		

C57BL/6 wild type mice	Charles River	C57BL/6J (JAX™ Mice Strain)

**Oligonucleotides**		

Mixl1-targetting guide pair 1 guide 1: AAGCGGCGCCTTCTGCGAAC	This paper	N/A
Mixl1-targetting guide pair 1 guide 2: TGCTGGGGCGCGAGAGTCGT	This paper	N/A
Mixl1-targetting guide pair 2 guide 1: TTGCGGCGCTGTGGCGCCGA	This paper	N/A
Mixl1-targetting guide pair 2 guide 2: CGCTCCCGCAAGTGGATGTC	This paper	N/A
primer including Nextera overhangs for guide pair 1: F- TCGTCGGCAGCGTCAGATGTGTATAAGAGACA GATTATTCCCGCGGCGTCT	This paper	N/A
primer including Nextera overhangs for guide pair 1: R- GTCTCGTGGGCTCGGAGATGTGTATAAGAGAC AGCTCCGAGCTGAACGACGT	This paper	N/A
primer including Nextera overhangs for guide pair 2: F- TCGTCGGCAGCGTCAGATGTGTATAAGAGACA GAGCAGCTCCAGTTCGCAGA	This paper	N/A
primer including Nextera overhangs for guide pair 2: R- GTCTCGTGGGCTCGGAGATGTGTATAAGAGAC AGCCCAGTTTGCAGTCTAGAACC	This paper	N/A

**Recombinant DNA**		

pX458 plasmid	Ran et al.^60^	Addgene, #48138

**Software and algorithms**		

*MouseGastrulationData* Bioconductor package	Griffiths^56^	https://bioconductor.org/packages/release/data/experiment/html/MouseGastrulationData.html
Cell Ranger v. 6.01	10x Genomics	N/A
R v.4.1.1	The R Project	https://www.r-project.org/
COSICC code	This paper	https://github.com/MarioniLab/analysis_chimera_data https://doi.org/10.5281/zenodo.15008255
COSICC package	This paper	https://github.com/MarioniLab/COSICC https://doi.org/10.5281/zenodo.15008255

**Other**		

Novaseq6000 sequencing platform	Illumina	N/A

### Experimental model and study participant details

#### Mouse embryonic stem cell lines

Mouse embryonic stem cell (mESC) lines were generated in-house and have not been authenticated. They were expanded under the 2i+LIF conditions,^58^ in a humidified incubator at 37°C and 7% CO2, and routinely tested negative for mycoplasma infection. A male, karyotypically normal, tdTomato-expressing mESC line^6^ was validated through ability to contribute to all embryo structures in a morula aggregation assay. It was then targeted to disrupt the *Mixl1* locus using the CRISPR/Cas9 system (see method details). Two mutant clones were used to generate *Mixl1*^−/−^ embryonic chimeras.

#### Mouse embryos

All procedures were performed in strict accordance with UK Home Office regulations for animal research. Chimeric mouse embryos were generated as described in the project licence number PPL 70/8406, following procedures detailed in Guibentif et al.^4^: animals used in this study were 6-10 week-old females, maintained on a lighting regime of 14 hours light and 10 hours darkness with food and water supplied *ad libitum*. For chimera generation, E3.5 blastocysts were derived from wildtype C57BL/6 matings, and after injection of the mutant cells, the resulting chimeric embryos were transferred to C57BL/6 recipient females at 0.5 days of pseudopregnancy following mating with vasectomised males. All chimeric embryos were collected at E8.5 (see details below).

### Method details

#### Mixl1^−/−^ embryo chimera data generation

All procedures involving mouse embryos were performed in strict accordance with the UK Home Office regulations for animal research under the project license number PPL 70/8406.

TdTomato-expressing mouse embryonic stem cells (ESC) were derived as previously described.^6^ Briefly, ESC lines were derived from E3.5 blastocysts obtained by crossing a male ROSA26tdTomato (Jax Labs – 007905) with a wildtype C57BL/6 female, expanded under the 2i+LIF conditions^59^ and transiently transfected with a Cre-IRES-GFP plasmid^60^ using Lipofectamine 3000 Transfection Reagent (ThermoFisher Scientific, #L3000008) according to manufacturer’s instructions. A tdTomato-positive, male, karyotypically normal line, competent for chimera generation as assessed using morula aggregation assay, was selected for targeting *Mixl1*. Two pairs of guides were designed to induce large deletions in Exon 2 of the *Mixl1* locus using the http://crispr.mit.edu tool (guide pair 1: AAGCGGCGCCTTCTGCGAAC and TGCTGGGGCGCGAGAGTCGT; guide pair 2: TTGCGGCGCTGTGGCGCCGA and CGCTCCCGCAAGTGGATGTC) and were cloned into the pX458 plasmid (Addgene, #48138) as previously described.^60^ The obtained plasmids were then used to transfect the cells and single transfected clones were expanded and assessed for Cas9-induced mutations. Genomic DNA was isolated by incubating cell pellets in 0.1 mg/ml of Proteinase K (Sigma, #03115828001) in TE buffer at 50°C for 2 hours, followed by 5 min at 99°C. The sequence flanking the guide-targeted sites was amplified from the genomic DNA by polymerase chain reaction (PCR) in a Biometra T3000 Thermocycler (30 sec at 98°C; 30 cycles of 10 sec at 98°C, 20 sec at 58°C, 20 sec at 72°C; and elongation for 7 min at 72°C) using the Phusion High-Fidelity DNA Polymerase (NEB, #M0530S) according to the manufacturer’s instructions. To assess the CRISPR/Cas9 targeting, primers to amplify the targeted regions and including Nextera overhangs were used (target pair 1: F- TCGTCGGCAGCGTCAGATGTGTATAAGAGACAGATTATTCCCGCGGCGTCT; R- GTCTCGTGGGCTCGGAGATGTGTATAAGAGACAGCTCCGAGCTGAACGACGT; target pair 2: F- TCGTCGGCAGCGTCAGATGTGTATAAGAGACAGAGCAGCTCCAGTTCGCAGA and R- GTCTCGTGGGCTCGGAGATGTGTATAAGAGACAGCCCAGTTTGCAGTCTAGAACC), allowing library preparation with the Nextera XT Kit (Illumina, #15052163), and sequencing was performed using the Illumina MiSeq system according to manufacturer’s instructions. One ESC clone from each guide pair transfection showing a frameshift mutation resulting in the functional inactivation of the *Mixl1* locus was selected for injection into C57BL/6 E3.5 blastocysts (Figure S5A). Chimeric embryos were harvested at E8.5, dissected, and single-cell suspensions were generated by TrypLE Express dissociation reagent (Thermo Fisher Scientific) incubation for 7-10 minutes at 37°C under agitation. Single-cell suspensions were sorted into tdTom+ and tdTom- samples using a BD Influx sorter with DAPI at 1μg/ml (Sigma) as a viability stain for subsequent 10X scRNA-seq library preparation (version 3 chemistry), and sequencing using the Illumina Novaseq6000 platform in one full S1 flowcell. In total, three independent pools of chimeric embryos were processed, two pools generated with a clone successfully targeted with guide pair 1 (containing respectively 3 and 6 embryos), and one clone successfully targeted with guide pair 2 (comprising 4 embryos). In the chimera cell pools, while all tdTom^+^ cells (progeny of the injected ESC lines) are male, the tdTom^-^ cells (progeny of host blastocysts) comprise a mix of both male and female cells. Thus to avoid sex bias in the results, *Xist* and Y chromosome genes were excluded for the highly variable genes during data processing, quality control (QC) and mapping (see section below on Data processing, QC and mapping to extended mouse gastrulation atlas) as in previous studies.^4^ COSICC also accounts for this potential sex bias, as it incorporates an external control (WT chimeras) with a similar sex bias (male injected cells, mixed host cells), so that perturbation effects identified are not driven by expression of sex genes.

This resulted in 17393 tdTom^-^ and 18754 tdTom^+^ cells that passed quality control (see “Data processing, QC and mapping to extended mouse gastrulation atlas” below), with an average of 2574 genes and 10727 UMIs detected per cell. To exclude transcriptional effects intrinsic to the chimera assay, chimeric embryos were generated by injecting the parental tdTom^+^ *Mixl1*^+/+^ (WT) line into C57BL/6 E3.5 blastocysts and processed as for the *Mixl1*^−/−^ samples.

### Quantification and statistical analysis

All the analyses and statistical tests were performed in R(v. 4.1.1). An R package was created to facilitate application of COSICC (https://github.com/MarioniLab/COSICC). All n values applied in the statistical analyses detailed below are listed in Table S2.

#### Data processing, QC and mapping to extended mouse gastrulation atlas

For the *Mixl1*^*−/−*^ chimeras, raw data were processed with Cell Ranger (v. 6.01, 10X Genomics) without mapping intronic reads, with reads mapped to the mm10 genome and counted with GRCm38.92 annotation to ensure consistency with the reference atlas^11^ and the existing *T*^*−/−*^ chimera data set.^4^ For the *T*^*−/−*^ chimera we used processed reads,^4^ for the *Mixl1*^−/−^ we followed the quality control steps detailed in Guibentif et al.^4^ and https://github.com/MarioniLab/EmbryoTimecourse2018 using R (v. 4.1.1).

#### COSICC overview

COSICC provides a comprehensive picture of knockout effects in complex organisms (Figure 1D). First, we investigated whether knockout causes differential abundance at the level of cell types, compared to changes seen between tdTom^+^ and tdTom^-^ cells from the WT chimeras (COSICC_DA_group, Figures 1F, 2A, and 3A). Second, for cell types represented at E9.25, we identified the cells comprising the inferred developmental trajectories ending in the respective cell types (henceforth called lineage trajectories), and tested whether a knockout would lead to abundance or depletion of cells for the lineage trajectory, again compared to changes in the WT chimeras (COSICC_DA_lineage, Figures 2C and 3B). Third, for lineage trajectories that were not severely depleted for knockout cells, we tested whether the progression along the lineage trajectory was delayed or accelerated (COSICC_kinetics, Figures 1G, 2D, 2E, 3C, 3D, S7D, and S7E). Finally, we applied a mixed effects model approach^10^ to test for differential gene expression for each cell type individually, again contrasted with external controls (WT chimeras, Figures 2F, 2G, and S5E).

#### COSICC_DA_group: group-based DA testing

COSICC_DA_group tests whether for a distinct group of cells (such as a cell type or sub-celltype) there is significant depletion or enrichment of tdTom^+^ cells compared to tdTom^-^ in the knockout chimeras, compared to the difference between tdTom^+^ and tdTom^-^ in the WT chimeras (Figure 1F).

Group-based DA testing (COSICC_DA_group) was performed as follows:

First, we discarded groups with less than 30 WT tdTom^+^ cells assigned to them. For each other group c the following ratios were computed:

$$r_{c,\text{target}}=\text{number}\;\text{of}\;{\text{tdTom}}^{+}\;\text{cells}\;\text{in}\;\text{knockout}\;\text{chimeras}\;\text{mapping}\;\text{to}\;c/\text{number}\;\text{of}\;{\text{tdTom}}^{-}\;\text{cells}\;\text{in}\;\text{knockout}\;\text{chimeras}\;\text{mapping}\;\text{to}\;c$$

$$r_{c,\text{control}}=\text{number}\;\text{of}\;{\text{tdTom}}^{+}\;\text{cells}\;\text{in}\;\text{WT}\;\text{chimeras}\;\text{mapping}\;\text{to}\;c/\text{number}\;\text{of}\;{\text{tdTom}}^{-}\;\text{cells}\;\text{in}\;\text{WT}\;\text{chimeras}\;\text{mapping}\;\text{to}\;c$$

As illustrated in Figure 1E, the data generation was affected by an experimental bias affecting compositional data analysis: groups that are not depleted will appear to be over-represented. We corrected for this sampling bias as follows (Figure 1F):

First we computed medians across all groups: m_target_ = median_c_(r_c,target_ ) and m_control_ = median_c_(r_c,control_), where median_c_ refers to the median across all groups.

Then we sampled for the knockout chimeras (without replacement) n_target_ cells among the tdTom^+^ cells and n_target_ x m_target_ cells among the tdTom^-^ cells, where n_target_ = min(number of tdTom^+^ cells in knockout chimera mapping to the group, number of tdTom^-^ cells in knockout chimera mapping to the group/m_target_). Under the assumption that most groups are not affected by depletion or enrichment as a result of the knockout (and that therefore the median ratio m_target_ is not affected), this normalisation corrects for the fact that given the depletion of larger groups, an approximately equal number of tdTom^+^ and tdTom^-^ cells overall will lead to spurious enrichment results for other groups, if not corrected. We proceeded analogously for the WT injected control chimeras.

After the sampling step described in the previous paragraph, we performed, for each group, a Fisher’s exact test to determine the odds ratio and p-value of a chimera cell mapping to a particular group being significantly more or significantly less likely to be tdTom^+^ than tdTom^-^. We repeated the sampling and the Fisher’s exact test 100 times to avoid dependence of the magnitude and significance of the inferred effect on the randomly chosen sample. For downstream analysis we used the median odds ratio and the median p-value, obtained as median across 100 subsamples.

The steps described above were applied to each group. FDR correction^61^ was subsequently applied across groups.

We applied COSICC_DA not only to cell types, but to a combination of cell type and the embryonic stage of a chimera cell (obtained from mapping to the reference atlas, see the section *Data processing, QC and mapping to extended mouse gastrulation atlas*), as described above with cell type and stage combinations replacing groups.

#### COSICC_DA_lineage: lineage trajectory-based DA testing

COSICC_DA_lineage tests whether tdTom^+^ chimera cells have an increased or decreased predisposition to develop into a particular cell type at a later stage (Figure 1C). COSICC_DA_lineage adapts COSICC_DA_group for lineage trajectory-based rather than cell type-based comparison by using the Waddington-OT method^9^ to obtain probabilities of a cell being part of a lineage trajectory. Waddington-OT, a method based on optimal transport, computes a matrix of transition probabilities linking cells at a specific time point to those at the next time point. Applying transition matrices repeatedly allows the computation of normalised fate matrices assigning to each cell and each lineage trajectory the probability that the cell is part of the specific lineage trajectory. For transitions between each pair of consecutive states, we used the Waddington-OT transition matrices for the reference atlas computed for publication of the extended mouse gastrulation atlas.^11^ To compute fate matrices from the transition matrices, we used the command line interface as in Section 5 of the Waddington-OT tutorial (https://broadinstitute.github.io/wot/tutorial/).

We included lineage trajectories whose respective terminal cell type contains more than 100 cells at E9.25 in the extended gastrulation atlas, and performed 10 iterations of the following:

For each cell from the chimeric embryos a lineage trajectory was sampled according to the probability distribution given by the Waddington-OT fate matrix. Given the sampled lineage trajectory for each cell (a fixed state and not a probability of being in a state), we applied COSICC_DA_group, performing the sampling of cells for sampling bias correction and Fisher tests as for COSICC_DA_group 30 times for each sampling of lineage trajectories based on the Waddington-OT fate matrix. We performed FDR correction across lineage trajectories.

We then computed the medians across odds-ratios and FDR-corrected p-values across the 10 repeats of sampling lineage trajectories based on the Waddington-OT fate matrix.

#### COSICC_kinetics: testing for acceleration or delay of knockout cells along a trajectory

To determine whether there is a delay or acceleration for tdTom^+^ cells along a trajectory, we combined Waddington-OT fate matrices with statistical modelling and pseudotime.

Cells were assigned to lineage trajectories using a mixture model of skewed t-distributions^62^ with two or three components (the optimal number of components was determined by the Bayesian information criterion.^63^ For each mapped stage, we proceeded as follows.

First we preselected those cells that had higher normalised Waddington-OT scores than random normalised Waddington-OT fate probabilities for the lineage trajectory (assuming a uniform random distribution), i.e. higher than the inverse of the total number of cells at their respective stage. Then, we fitted the mixture model and assigned to the lineage trajectory those cells that were in the cluster of the highest Waddington-OT fate probabilities. For each stage we discarded cell types that did not constitute at least 10% of the cells for the lineage trajectory at that stage, to mitigate any potential mistakes from the algorithm used to map the chimera data to the reference data set influencing the trajectory. From the time point onward from which the final cell type was the most frequent cell type in a lineage trajectory we excluded all other cell types from the lineage trajectory.

For the reference data set, the extended mouse gastrulation atlas, temporal genes were selected by testing their association with embryonic time using ANOVA (reference-based temporal genes). Genes for which the residuals from the ANOVA regression were associated with batch, i.e. genes correlated with time to different degrees for different batches, were removed from the list of reference-based temporal genes. Then we computed diffusion maps for the reference atlas cells^64^^,^^65^ based on the reference-based temporal genes to obtain a more fine-grained resolution of temporal development. We identified pseudotime as the diffusion component most correlated with actual time, to avoid focusing on components not related to time, but to e.g. spatial location. As the starting point for the trajectories we generally used E7.5, as most chimeric tdTom^+^ and tdTom^-^ cells mapped between E7.75 and E9.0 (Figure S1D). COSICC_kinetics was applied to all lineage trajectories that were not severely depleted for tdTom^+^ chimera cells, i.e. for which the odds ratio of (tdTom^+^ cells for knockout chimera/tdTom^-^ cells for knockout chimeras)/(tdTom^+^ cells for WT chimera/tdTom^-^ cells for WT chimeras) > 0.05.

Pseudotimes were assigned to chimera cells by assigning to the chimera cell the average of the pseudotimes of the 10 atlas cells to which the chimera cell was most correlated, where for the computation of correlation we used the reference-based temporal genes only. This ensured that the pseudotime mapping was based on genes that differ across stages in the reference data set and excluded genes whose expression depended on batch.

We used the Wilcoxon rank-sum test^66^ to identify trajectories whose median was more strongly shifted in tdTom^+^ cells for the knockout chimeras than for the WT chimeras (Figures 1G, 2E, 3C, S7D and S7E). This identified trajectories for which the difference between tdTom^+^ and tdTom^-^ cells for the knockout chimeras was significant and the 95% confidence intervals for the location parameter (the difference in pseudotime median for tdTom^+^ versus tdTom^-^ cells) of the respective Wilcoxon tests were non-overlapping between WT and knockout chimeras.

#### DE testing for chimera data

DE tests were performed for each cell type separately. To test for DE between tdTom^+^ and tdTom^-^ cells in the knockout chimeras, contrasted with the same change in the WT chimeras, while also accounting for batch effects across the different pools of chimeras, we used the following negative binomial mixed effects model, applying the NEBULA^10^ approach:

$$y_{ij}∼NB\left(\mu_{ij}=\pi_{j}\cdot exp\left(\alpha_{i}+b_{1,i}\cdot m_{j}+b_{2,i}\cdot \;t_{j}+b_{3,i}\cdot m_{j}\cdot t_{j}+\log\left(w_{i}|p_{j}\right)\right),\phi_{i}\right)$$

where $y_{ij}$ is the expression of gene *i* in cell *j*, $m_{j}$ is binary with $m_{j}=1$ if the cell is marked by the fluorescent marker (i.e. a tdTom^+^ cell), $t_{j}=1$ if the cell is part of the knockout chimeras (as opposed to the control chimeras), $\pi_{j}$ is the size-factor of cell j (computed using the scran^67^ Bioconductor package as part of normalisation) and $\phi_{i}$ is the dispersion parameter for the negative Binomial distribution for gene *i*. $w_{i}$ is a random effect at the level of the pool p_j_ containing cell j. We then test whether b_3,i_ is significantly different from 0, as this means a significant difference between the knockout and WT chimeras.

#### Treatment of batch effects across pools of chimeras

Chimeras were obtained in pools of several embryos (see Section *Mixl1*^*-/-*^ *embryo chimera data generation*), without information concerning individual embryos. Therefore, we were not able to model the effect of inter-embryo variation. We checked consistency across pools for DA testing (Figure S2), reduced the potential impact of batch effects across pools for COSICC_DA_kinetics by computing pseudotimes based on reference-based temporal genes (see Section *COSICC_kinetics: testing for acceleration or delay of knockout cells along a trajectory*), and explicitly modelled the batch structure for DE testing using mixed effects models.

#### JCF scores

JCF scores were obtained by averaging the expression levels of JCF genes (https://crukci.shinyapps.io/heartAtlas/, cluster marker genes for me5) obtained from the single-cell atlas of the mouse embryonic heart.^30^

#### Sub-clustering of mesenchyme cell type

We used Louvain clustering^68^ on a nearest neighbour graph with k=20 neighbours. To enable detection of genes differentially expressed across sub-clusters without inflation of the false positive rate, we used only 50% of the cells in the clustering algorithm and assigned the remaining cells to the clusters using support vector machines.^69^ We then applied COSICC_DA_group to the subgroups of the mesenchyme cell type in the same way as to cell types.

## Supplemental information

Document S1. Figures S1–S7

Table S1. Results from analysis of T−/− and Mixl1−/− datasets

Table S2. All n values in statistical analyses relevant for Figures 2 and 3
