# Supplementary material for: A single-cell and tissue-scale analysis suite resolves Mixl1’s role in heart development
Source: iScience. 2025 Apr 10;28(5):112397. doi: 10.1016/j.isci.2025.112397 (PMC12051648; doi:10.1016/j.isci.2025.112397)
Supplement: Document S1. Figures S1–S7 [file mmc1.pdf]

**iScience, Volume *28***

## Supplemental information

# A single-cell and tissue-scale analysis suite resolves Mixl1's role in heart development

**Magdalena E. Strauss, Mai-Linh Nu Ton, Samantha Mason, Jaana Bagri, Luke T.G. Harland, Ivan Imaz-Rosshandler, Nicola K. Wilson, Jennifer Nichols, Richard C.V. Tyser, Berthold Göttgens, John C. Marioni, and Carolina Guibentif**

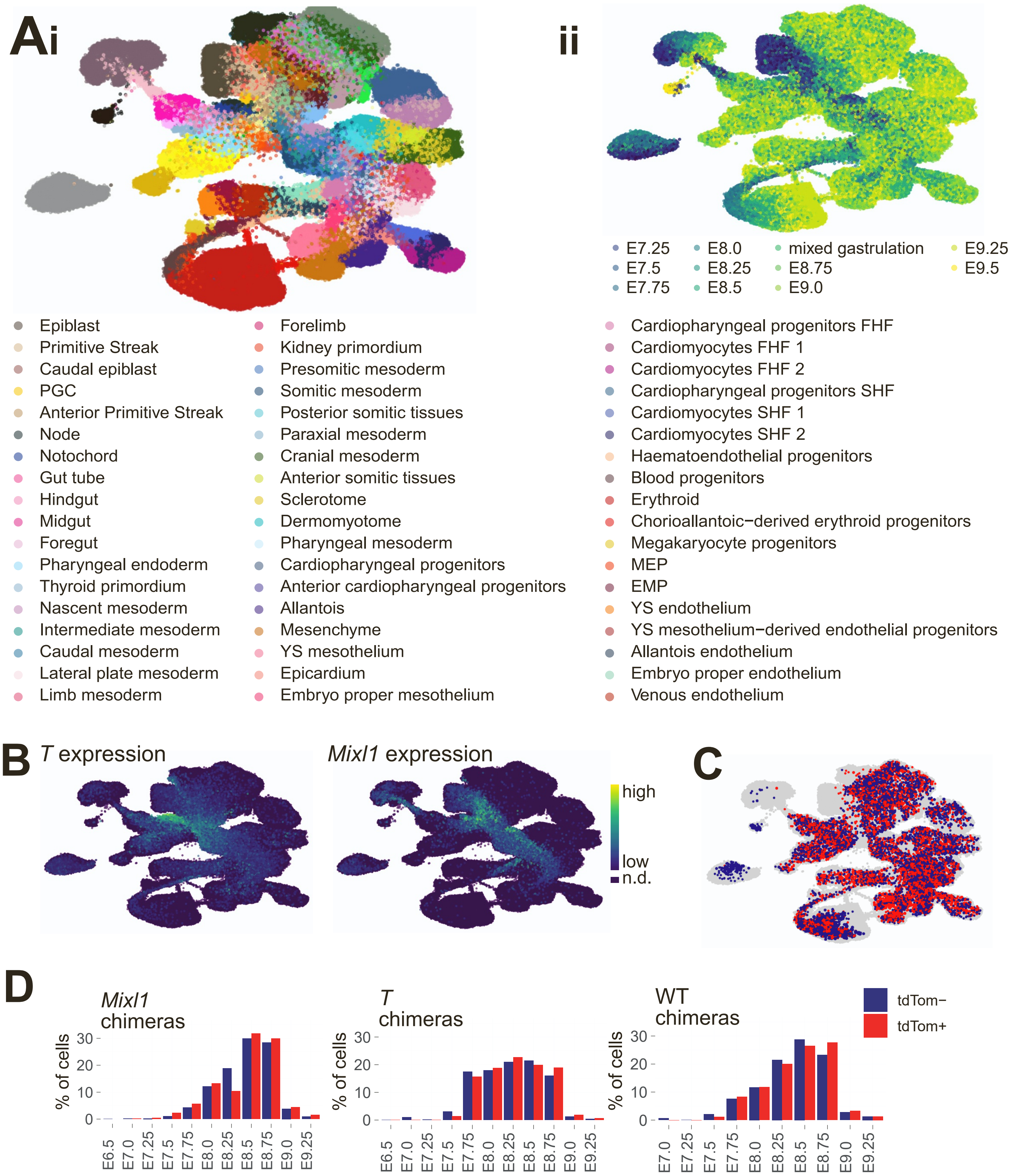
Ai
ii
E7.25
E7.5
E7.75
E8.0
E8.25
E8.5
mixed gastrulation
E8.75
E9.0
E9.25
E9.5
Epiblast
Primitive Streak
Caudal epiblast
PGC
Anterior Primitive Streak
Node
Notochord
Gut tube
Hindgut
Midgut
Foregut
Pharyngeal endoderm
Thyroid primordium
Nascent mesoderm
Intermediate mesoderm
Caudal mesoderm
Lateral plate mesoderm
Limb mesoderm
Forelimb
Kidney primordium
Presomitic mesoderm
Somitic mesoderm
Posterior somitic tissues
Paraxial mesoderm
Cranial mesoderm
Anterior somitic tissues
Sclerotome
Dermomyotome
Pharyngeal mesoderm
Cardiopharyngeal progenitors
Anterior cardiopharyngeal progenitors
Allantois
Mesenchyme
YS mesothelium
Epicardium
Embryo proper mesothelium
Cardiopharyngeal progenitors FHF
Cardiomyocytes FHF 1
Cardiomyocytes FHF 2
Cardiopharyngeal progenitors SHF
Cardiomyocytes SHF 1
Cardiomyocytes SHF 2
Haematoendothelial progenitors
Blood progenitors
Erythroid
Chorioallantoic−derived erythroid progenitors
Megakaryocyte progenitors
MEP
EMP
YS endothelium
YS mesothelium−derived endothelial progenitors
Allantois endothelium
Embryo proper endothelium
Venous endothelium
B
T expression
Mixl1 expression
high
low
n.d.
C
D
Mixl1 chimeras
T chimeras
WT chimeras
% of cells
0
10
20
30
E6.5
E7.0
E7.25
E7.5
E7.75
E8.0
E8.25
E8.5
E8.75
E9.0
E9.25
tdTom−
tdTom+

**Figure S1 (related to Figure 1): Modelling based on the extended mouse gastrulation atlas.**
A) UMAP representation of extended mouse gastrulation atlas [S1], coloured by cell types (i) and stages (ii) B) Expression of *T* and *Mixl1* in the extended mouse gastrulation atlas. n.d.: not detected. C) Mapping of the WT chimeras to the extended mouse gastrulation data set illustrates differences between injected tdTom$^{+}$ (red) and host tdTom$^{-}$ (blue) cells. D) Mapping of cells from chimeras harvested at E8.5 to different stages in the extended mouse gastrulation atlas. The actual time of collection is E8.5, but as expected we observe differences in developmental progression within the embryo litters. Thus some cells are delayed in development (earlier than E8.5), and others advanced.

A

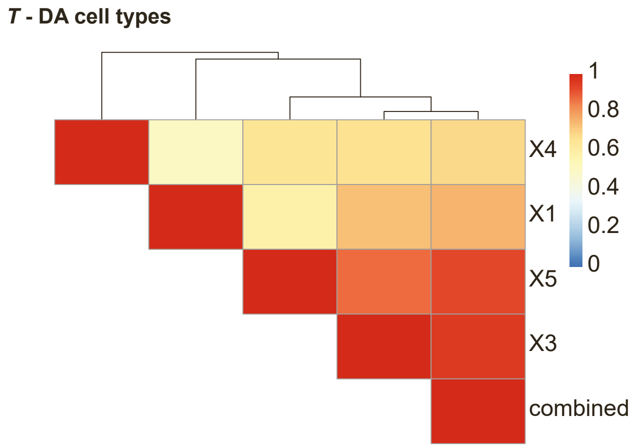

T - DA cell types
X4
X1
X5
X3
combined
1
0.8
0.6
0.4
0.2
0


B

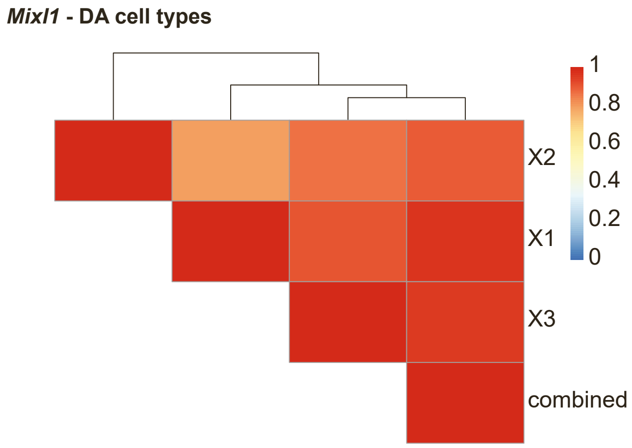

Mixl1 - DA cell types
X2
X1
X3
combined
1
0.8
0.6
0.4
0.2
0

**Figure S2 (related to Methods): Spearman rank correlation between COSICC_DA results based on individual pools of chimeras.**

A) Correlation between COSICC_DA results in the individual pools (X1, X3, X4, X5) of $T^{-/-}$ chimeras as well as in all pools combined, based on all cell types that were found to be significantly DA for at least one of the pools or the combined data set. B) As A), for *Mixl1*$^{-/-}$ chimeras. A) and B) The high levels of correlation highlight the concordance of the results across the independent chimeric embryo pools. See Table S2 for cell numbers for cell type and sample.

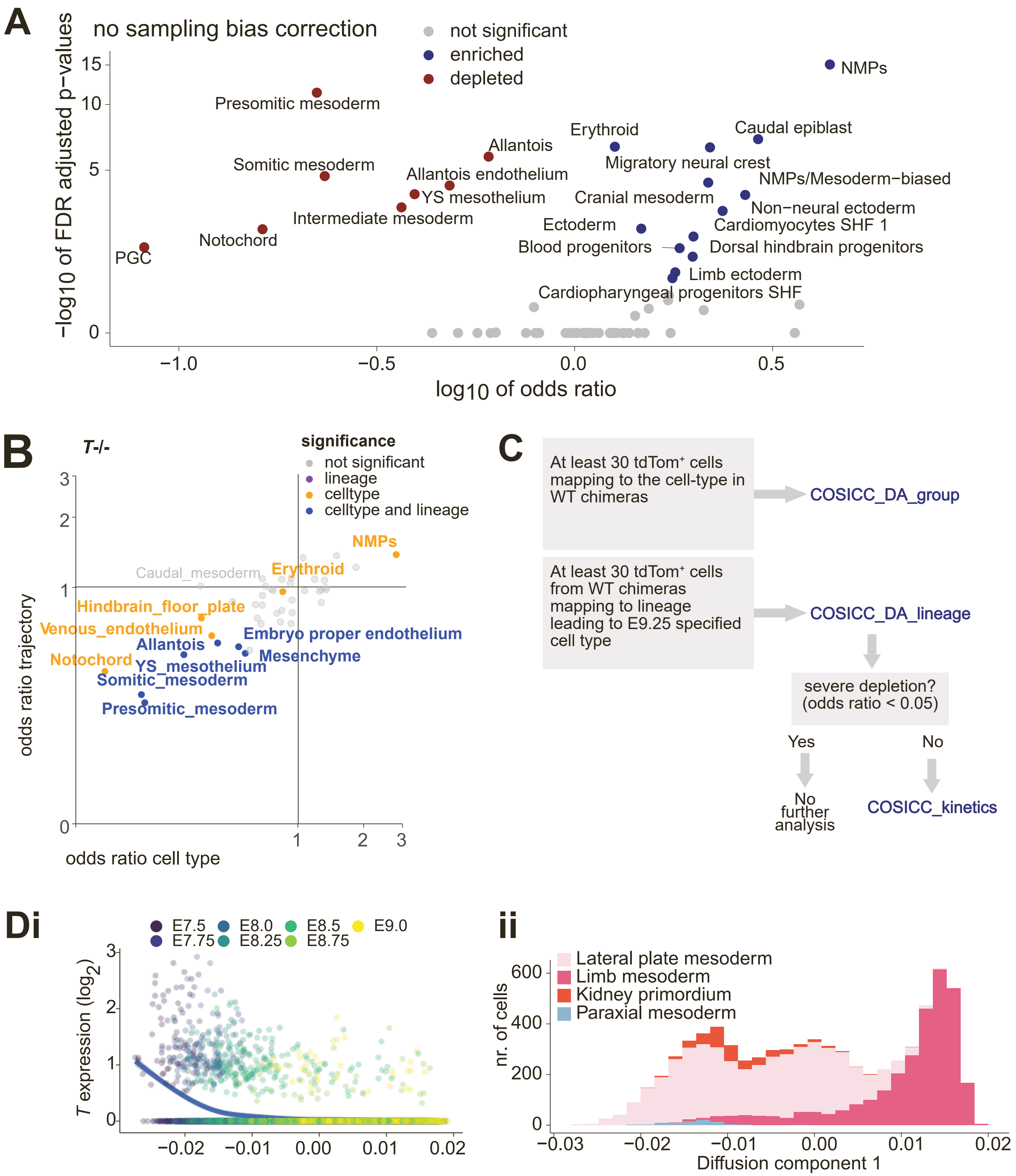
A
no sampling bias correction
not significant
enriched
depleted
-log10 of FDR adjusted p-values
log10 of odds ratio
NMPs
Presomitic mesoderm
Erythroid
Caudal epiblast
Allantois
Somitic mesoderm
Allantois endothelium
Migratory neural crest
NMPs/Mesoderm−biased
YS mesothelium
Cranial mesoderm
Non−neural ectoderm
Intermediate mesoderm
Ectoderm
Cardiomyocytes SHF 1
Notochord
Blood progenitors
Dorsal hindbrain progenitors
PGC
Limb ectoderm
Cardiopharyngeal progenitors SHF
B
T-/-
significance
not significant
lineage
celltype
celltype and lineage
NMPs
Caudal_mesoderm
Erythroid
Hindbrain_floor_plate
Venous_endothelium
Embryo proper endothelium
Allantois
Mesenchyme
Notochord
YS_mesothelium
Somitic_mesoderm
Presomitic_mesoderm
odds ratio trajectory
odds ratio cell type
C
At least 30 tdTom+ cells mapping to the cell-type in WT chimeras
COSICC_DA_group
At least 30 tdTom+ cells from WT chimeras mapping to lineage leading to E9.25 specified cell type
COSICC_DA_lineage
severe depletion? (odds ratio < 0.05)
Yes
No
No further analysis
COSICC_kinetics
Di
E7.5
E7.75
E8.0
E8.25
E8.5
E8.75
E9.0
T expression (log2)
ii
Lateral plate mesoderm
Limb mesoderm
Kidney primordium
Paraxial mesoderm
nr. of cells
Diffusion component 1

**Figure S3: DA analysis (related to Figure 2 and Figure 3).**

A) DA testing using Fisher's exact test (i.e. identical to COSICC_DA but without the sampling bias correction step) leads to spurious enrichment results. B) Comparing lineage-based to cell type-based enrichment and depletion of $T^{-/-}$ cells. C) Schematic of the applicability of COSICC_DA_group, COSICC_DA_lineage and COSICC_kinetics, which rely on sufficient numbers of cells being present in the analysed cell type/lineage. See also Figure S4. D) i) Expression of *T* for the limb mesoderm lineage. The line shows smoothed gene expression (Loess curve). ii) Distribution of cell types along the limb mesoderm lineage for unperturbed extended mouse gastrulation atlas cells, with progression along the diffusion component most correlated with embryonic time (Diffusion component 1). See Table S2 for cell numbers for each cell type and sample.

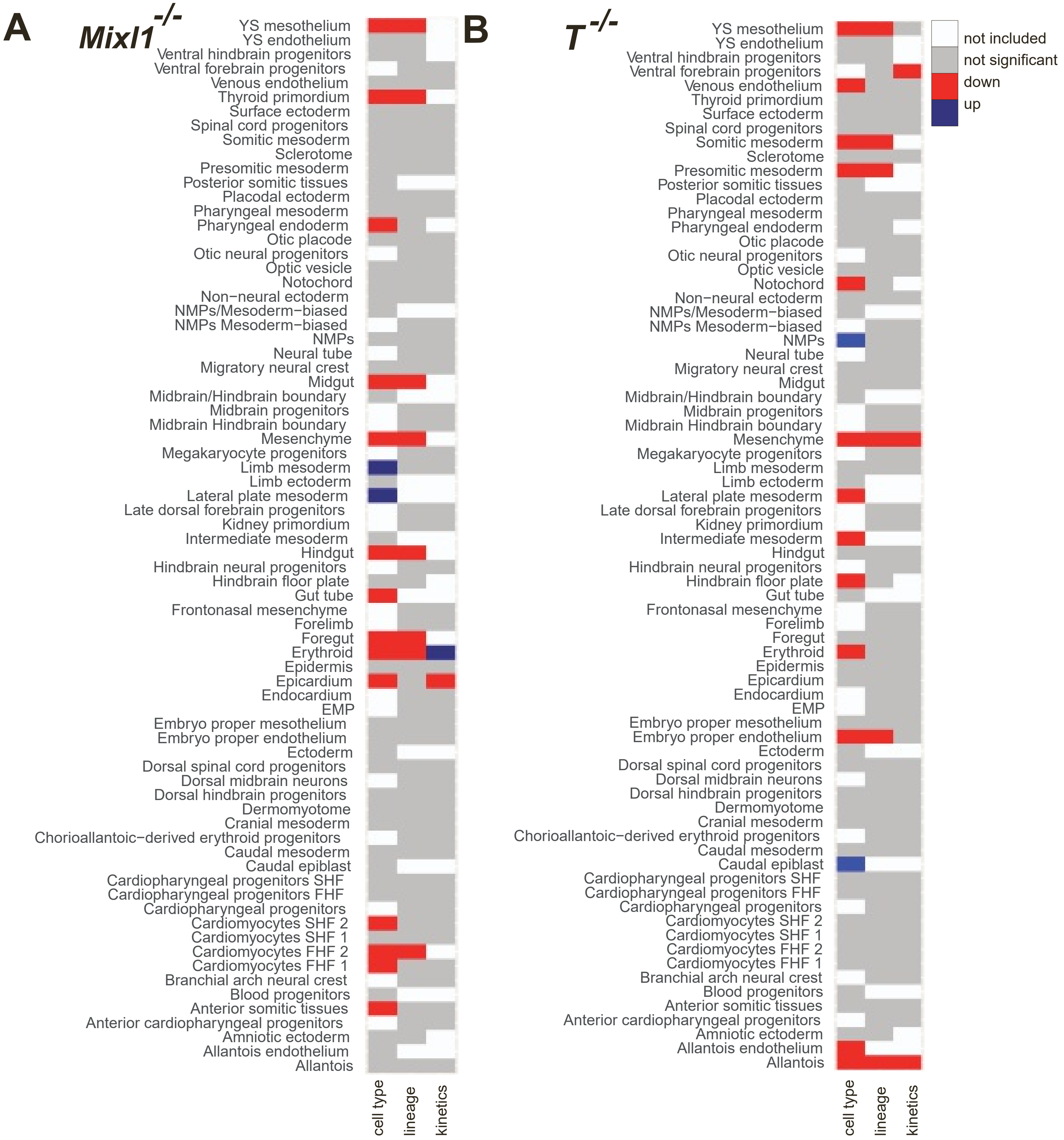
A
Mixl1-/-
B
T-/-
YS mesothelium
YS endothelium
Ventral hindbrain progenitors
Ventral forebrain progenitors
Venous endothelium
Thyroid primordium
Surface ectoderm
Spinal cord progenitors
Somitic mesoderm
Sclerotome
Presomitic mesoderm
Posterior somitic tissues
Placodal ectoderm
Pharyngeal mesoderm
Pharyngeal endoderm
Otic placode
Otic neural progenitors
Optic vesicle
Notochord
Non−neural ectoderm
NMPs/Mesoderm−biased
NMPs Mesoderm−biased
NMPs
Neural tube
Migratory neural crest
Midgut
Midbrain/Hindbrain boundary
Midbrain progenitors
Midbrain Hindbrain boundary
Mesenchyme
Megakaryocyte progenitors
Limb mesoderm
Limb ectoderm
Lateral plate mesoderm
Late dorsal forebrain progenitors
Kidney primordium
Intermediate mesoderm
Hindgut
Hindbrain neural progenitors
Hindbrain floor plate
Gut tube
Frontonasal mesenchyme
Forelimb
Foregut
Erythroid
Epidermis
Epicardium
Endocardium
EMP
Embryo proper mesothelium
Embryo proper endothelium
Ectoderm
Dorsal spinal cord progenitors
Dorsal midbrain neurons
Dorsal hindbrain progenitors
Dermomyotome
Cranial mesoderm
Chorioallantoic−derived erythroid progenitors
Caudal mesoderm
Caudal epiblast
Cardiopharyngeal progenitors SHF
Cardiopharyngeal progenitors FHF
Cardiopharyngeal progenitors
Cardiomyocytes SHF 2
Cardiomyocytes SHF 1
Cardiomyocytes FHF 2
Cardiomyocytes FHF 1
Branchial arch neural crest
Blood progenitors
Anterior somitic tissues
Anterior cardiopharyngeal progenitors
Amniotic ectoderm
Allantois endothelium
Allantois
cell type
lineage
kinetics
not included
not significant
down
up

**Figure S4 (related to Figures 2 and 3): Effect of gene knockouts on all cell types for *Mixl1*$^{-/-}$ (A) and *T*$^{-/-}$ (B) chimeras.** Grey colour signifies that a test was performed, but there was no significant enrichment or depletion. White signifies that the test was not performed due to insufficient representation of tdTom$^{+}$ fractions (see Figure S3C for applicability of COSICC analyses).

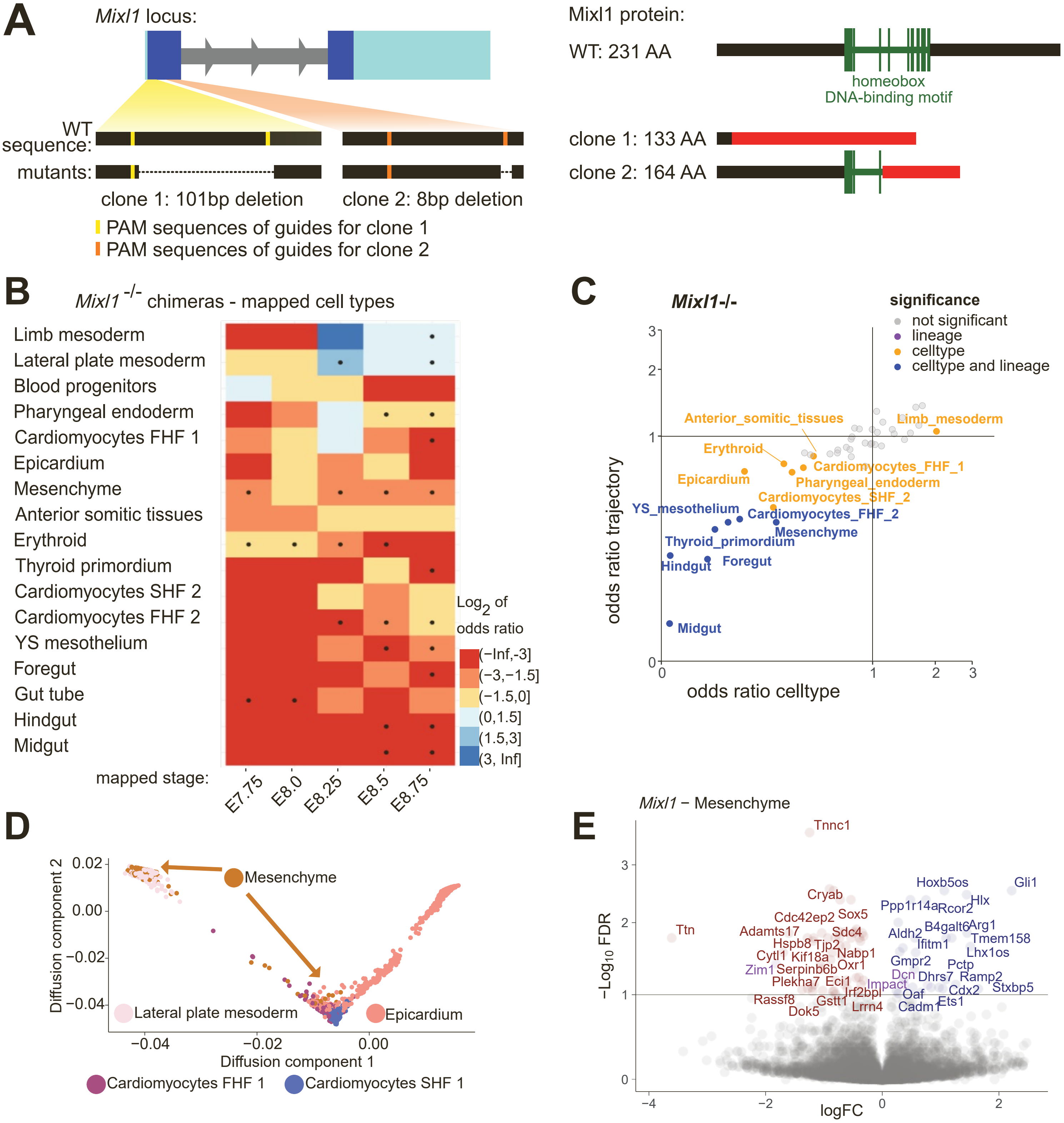
A
Mixl1 locus:
WT sequence:
mutants:
clone 1: 101bp deletion
clone 2: 8bp deletion
PAM sequences of guides for clone 1
PAM sequences of guides for clone 2
Mixl1 protein:
WT: 231 AA
homeobox DNA-binding motif
clone 1: 133 AA
clone 2: 164 AA
B
Mixl1 -/- chimeras - mapped cell types
Limb mesoderm
Lateral plate mesoderm
Blood progenitors
Pharyngeal endoderm
Cardiomyocytes FHF 1
Epicardium
Mesenchyme
Anterior somitic tissues
Erythroid
Thyroid primordium
Cardiomyocytes SHF 2
Cardiomyocytes FHF 2
YS mesothelium
Foregut
Gut tube
Hindgut
Midgut
mapped stage:
E7.75
E8.0
E8.25
E8.5
E8.75
Log2 of odds ratio
(−Inf,−3]
(−3,−1.5]
(−1.5,0]
(0,1.5]
(1.5,3]
(3, Inf]
C
Mixl1-/-
significance
not significant
lineage
celltype
celltype and lineage
Anterior_somitic_tissues
Limb_mesoderm
Erythroid
Cardiomyocytes_FHF_1
Epicardium
Pharyngeal_endoderm
Cardiomyocytes_SHF_2
YS_mesothelium
Cardiomyocytes_FHF_2
Mesenchyme
Thyroid_primordium
Hindgut
Foregut
Midgut
odds ratio trajectory
odds ratio celltype
D
Mesenchyme
Lateral plate mesoderm
Epicardium
Cardiomyocytes FHF 1
Cardiomyocytes SHF 1
Diffusion component 1
Diffusion component 2
E
Mixl1 − Mesenchyme
Tnnc1
Cryab
Ttn
Cdc42ep2
Sox5
Adamts17
Sdc4
Hspb8
Tjp2
Cytl1
Kif18a
Nabp1
Zim1
Serpinb6b
Oxr1
Plekha7
Eci1
Irf2bpl
Impact
Rassf8
Gstt1
Lrrn4
Dok5
Hoxb5os
Gli1
Ppp1r14a
Rcor2
Hlx
B4galt6
Arg1
Aldh2
Ifitm1
Tmem158
Lhx1os
Gmpr2
Pctp
Dcn
Dhrs7
Ramp2
Oaf
Cdx2
Stxbp5
Cadm1
Ets1
logFC
−Log10 FDR

**Figure S5 (related to Figure 3): *Mixl1$^{-/-}$* chimera generation and analysis.**
A) Left: schematic of the *Mixl1* locus, with the CRISPR/Cas9 deletions leading to the generation of each of the *Mixl1* mutant ESC clones. PAM: protospacer adjacent motif. Right: schematic of the Mixl1 protein, with the effects of the CRISPR/Cas9 deletions on the resulting protein in the mutant ESC clones. Altered protein sequences resulting from the frameshift mutations are depicted in red. B) DA per mapped stage and cell type for *Mixl1$^{-/-}$* chimeras. C) Comparing lineage-based to cell type-based enrichment and depletion of *Mixl1$^{-/-}$* cells. D) Epicardium lineage from E7.5 to E9.0 in the extended mouse gastrulation atlas, coloured by cell type. E) Volcano plot illustrating statistically differentially expressed genes for mesenchyme. The x-axis represents the log2-fold change for differential expression contrasted with the WT chimeras. See Table S2 for cell numbers for each stage (mapped from the extended mouse gastrulation atlas), cell type and sample.

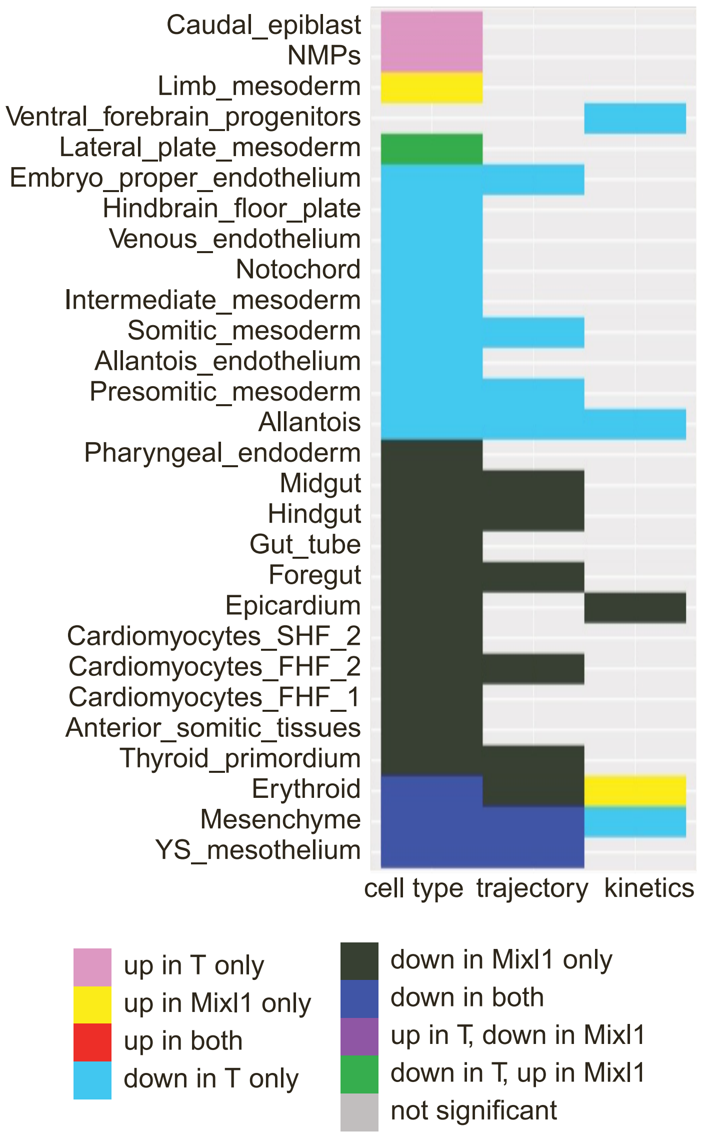

Caudal_epiblast
NMPs
Limb_mesoderm
Ventral_forebrain_progenitors
Lateral_plate_mesoderm
Embryo_proper_endothelium
Hindbrain_floor_plate
Venous_endothelium
Notochord
Intermediate_mesoderm
Somitic_mesoderm
Allantois_endothelium
Presomitic_mesoderm
Allantois
Pharyngeal_endoderm
Midgut
Hindgut
Gut_tube
Foregut
Epicardium
Cardiomyocytes_SHF_2
Cardiomyocytes_FHF_2
Cardiomyocytes_FHF_1
Anterior_somitic_tissues
Thyroid_primordium
Erythroid
Mesenchyme
YS_mesothelium
cell type
trajectory
kinetics
up in T only
up in Mixl1 only
up in both
down in T only
down in Mixl1 only
down in both
up in T, down in Mixl1
down in T, up in Mixl1
not significant

**Figure S6 (related to Figures 2 and 3): Across-chimera comparison.** Comparing effects between *T*$^{-/-}$ and *Mixl1*$^{-/-}$, for DA of cell type, DA of lineage and differential speed of progression along the trajectory.

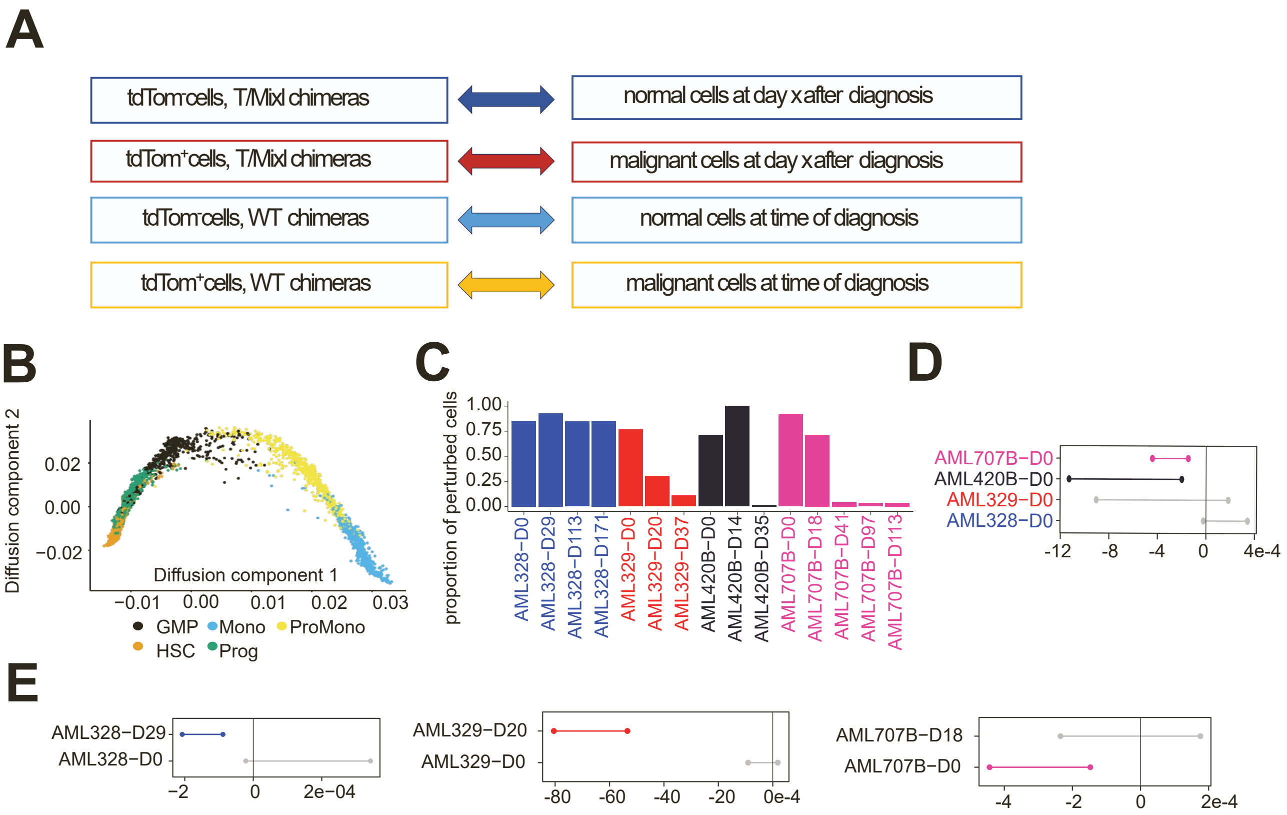

A
tdTom⁻cells, T/Mixl chimeras
normal cells at day x after diagnosis
tdTom⁺cells, T/Mixl chimeras
malignant cells at day x after diagnosis
tdTom⁻cells, WT chimeras
normal cells at time of diagnosis
tdTom⁺cells, WT chimeras
malignant cells at time of diagnosis
B
Diffusion component 2
Diffusion component 1
GMP Mono ProMono
HSC Prog
C
proportion of perturbed cells
AML328−D0
AML328−D29
AML328−D113
AML328−D171
AML329−D0
AML329−D20
AML329−D37
AML420B−D0
AML420B−D14
AML420B−D35
AML707B−D0
AML707B−D18
AML707B−D41
AML707B−D97
AML707B−D113
D
AML707B−D0
AML420B−D0
AML329−D0
AML328−D0
E
AML328−D29
AML328−D0
AML329−D20
AML329−D0
AML707B−D18
AML707B−D0

**Figure S7 (related to Figure 1): Application of COSICC to gain insights into Acute Myeloid Leukemia progression.**

A) COSICC was applied to a published AML data set [S2] using internal and external controls equivalent to the chimera set-up: D0 samples correspond to WT chimeras, later day samples to knockout chimeras. Within these samples, malignant cells correspond to the tdTom+ fraction in our chimera study and normal cells correspond to the tdTom- cells. Healthy bone-marrow was used as a reference in lieu of the extended mouse gastrulation atlas. B) Diffusion components for myeloid trajectory in healthy bone marrow reference single-cell data set, based on genes associated with development along the myeloid trajectory but without significant differences between donors (in the chimeras these would correspond to the temporal genes identified in the mouse gastrulation atlas (Methods)). Transcriptomes are coloured by cell type as annotated in [S2]. C) and D) Patients with less than 95% malignant cells at D0 (diagnosis): C) Proportion of malignant cells along the myeloid trajectory. D) Wilcoxon rank-sum test for delay along the myeloid trajectory for malignant cells compared to their normal counterparts shows a significant delay for patients AML-707B and AML-420B at day 0 (COSICC_kinetics, 95% confidence intervals (bars) below and not crossing 0), suggesting a stem cell-like phenotype of malignant cells. E) COSICC_kinetics test for significance of changes in the delay over time (requires a percentage of malignant cells in the myeloid trajectory between 5% and 95% at the first time point after D0). Application of COSICC to this AML data set illustrates the method’s wide range of applicability and highlights the importance of incorporating appropriate controls to extract meaningful insights from clinical single-cell data [S3]. See Table S2 with n values corresponding to the number of cells for each patient and time point.

**Supplemental References**

S1. Imaz-Rosshandler, I., et al., Tracking early mammalian organogenesis - prediction and validation of differentiation trajectories at whole organism scale. *Development*, 2024. **151**(3).

S2. van Galen, P., et al., Single-Cell RNA-Seq Reveals AML Hierarchies Relevant to Disease Progression and Immunity. *Cell*, 2019. **176**(6): p. 1265-1281 e24.

S3. Sheridan, C. (2024). Can single-cell biology realize the promise of precision medicine? *Nat. Biotechnol.* **42**, p. 159-162.
